# The effect of yin yoga intervention on state and trait anxiety during the COVID-19 pandemic

**DOI:** 10.3389/fpsyt.2024.1345455

**Published:** 2024-03-14

**Authors:** Kristīne Somere, Maris Munkevics, Ronalds Krams, Gunta Rača, Severi Luoto, Indrikis Krams

**Affiliations:** ^1^ Department of Human and Animal Physiology, University of Latvia, Riga, Latvia; ^2^ Department of Zoology and Animal Ecology, University of Latvia, Riga, Latvia; ^3^ Statistics Unit, Riga Stradins University, Riga, Latvia; ^4^ Sport Center, University of Latvia, Riga, Latvia; ^5^ Institute of Life Sciences and Technologies, Daugavpils University, Daugavpils, Latvia; ^6^ School of Population Health, University of Auckland, Auckland, New Zealand; ^7^ Latvian Biomedicine Research and Study Centre, Riga, Latvia

**Keywords:** Yin yoga, state anxiety, trait anxiety, COVID-19, stress

## Abstract

**Introduction:**

Although some findings indicate that yoga can reduce stress and anxiety, many studies present mixed results. The potential of yoga interventions to alleviate anxiety, including the mechanisms and boundary conditions by which it does so, is an under-researched topic. Anxiety is often divided into “state anxiety” and “trait anxiety,” the former being a temporary reaction to stressful events, while the latter is a more stable personality feature that responds to adverse situations or perceived threats.

**Materials and methods:**

This study investigates whether a yin yoga intervention delivered online reduces state anxiety immediately after each yoga session and whether the anxiety levels are significantly lower at the end of the 10-week yoga intervention than at the beginning of the study. We also predicted no effect of yin yoga intervention on trait anxiety. The study was conducted during the COVID-19 pandemic when participants (*N* = 48 Latvian women) experienced heightened anxiety levels.

**Results:**

This study shows that a ten-week online yin yoga intervention significantly reduced state anxiety in the intervention group compared with the control group. State anxiety levels also significantly decreased after *each* yin yoga session, providing more support for the anxiety-reducing effect of yin yoga. In contrast, yoga participation did not cause differences in trait anxiety between the control and intervention groups, even though trait anxiety decreased in the intervention group and increased in the control group over the study period.

**Conclusion:**

The positive effects of yin yoga on state anxiety indicate the potential of yin yoga intervention as a first-line treatment to control and reduce state anxiety, with possible additional effects on trait anxiety.

## 1 Introduction

Anxiety is a psychological, physiological, and behavioral state induced by a threat to emotional security, well-being, and survival, either actual or potential (1). It is characterized by increased arousal, expectancy, autonomic and neuroendocrine activation, and specific behavior patterns (2). The primary function of fear and anxiety is to act as a signal of danger, threat, or motivational conflict and to trigger appropriate adaptive responses (3). The function of anxiety-related behavioral reactions is to facilitate coping with an adverse or unexpected situation. However, pathological anxiety interferes with successfully dealing with life challenges (4).

The contemporary increase in anxiety levels may arise because of a mismatch between the current environment and the environment of evolutionary adaptiveness (5). Modern people deal with many more everyday tasks and stimuli than they adapted to during their evolutionary history. For example, the numerous tasks and societal complexity faced by contemporary people may make many people less efficient and more stressed (6). Constantly high anxiety levels increase the vulnerability to psychopathology, depending on predisposing factors resulting from gene-environment interactions. Thus, anxiety, often an adaptive response driving coping behaviors to face possible dangers, can become dysfunctional if it is excessive and unmotivated, paving the way for developing anxiety disorders, clinical depression, bipolar disorders, and schizophrenia (7–9).

The COVID-19 pandemic has affected the physical and mental health of many people (10) and increased the number of individuals seeking medical and psychological help for their anxiety disorders, suggesting that the need for effective anxiety management has been exceptionally high during the pandemic (11). Many people had to completely change their work routine when remote work became the norm for office workers, students, and teachers alike during the COVID-19 pandemic. Many people, particularly women, had to combine their professional work with household duties and, in some cases, looking after children as well. This created a potentially stressful working environment with no clear boundary between work and domestic life. A study conducted in Turkey during a COVID-19 lockdown in 2020 reported that poor sleep quality, increased workload, and being female predicted increased anxiety among remote workers during the pandemic, while poor sleep quality, trouble focusing at work, being female, having financial concerns, and experiencing workplace loneliness predicted stress among remote workers (12). Other research with Israeli participants reported that remote work during the COVID-19 pandemic was significantly associated with anxiety among men and with depression among women. Increased anxiety and depressive symptomatology brought about by pandemic-related occupational changes, such as having to work remotely from home, comprises a potential risk factor for occupational wellbeing (13, 14).

Besides pharmaceutical treatment, it has been demonstrated that physical and mental exercise, including yoga, can reduce anxiety levels and improve psychological well-being (15–19). Yoga offers a popular and promising but underrated approach to reduce anxiety (20–23). Traditional forms of yoga incorporate physical postures and exercises, breath regulation, relaxation, meditation, and mindfulness practice. The popularity of yoga has markedly increased during the COVID-19 pandemic because it offered a physical exercise routine and a mental health facilitator to cope with the pandemic period and the societal measures that were implemented to curb transmission of the virus (24).

In anxiety, the sympathetic nervous system, responsible for the fight or flight response, operates in overdrive. As a consequence, the parasympathetic nervous system is underactive. The vagus nerve represents the main component of the parasympathetic nervous system, which oversees a vast array of crucial bodily functions, including control of mood, immune response, digestion, and heart rate (25). It establishes one of the connections between the brain and the gastrointestinal tract and supplies information about the state of the inner organs to the brain. There is preliminary evidence that vagus nerve stimulation is a promising add-on treatment for depression, post-traumatic stress disorder, and inflammatory bowel disease. Therapies that target the vagus nerve increase the vagal tone and inhibit proinflammatory cytokine production (26). Since the vagal tone is correlated with the capacity to regulate stress responses and can be influenced by breathing, its increase through meditation and yoga likely contributes to resilience and the mitigation of mood and anxiety symptoms (23). Thus, practicing yoga is an effective way to stimulate the vagus nerve and potentially decrease anxiety. However, meta-analyses on the effect of yoga on anxiety have yielded mixed results (21, 22).

Anxiety has been traditionally divided into “state anxiety” and “trait anxiety” (27, 28). To better understand anxiety as well as its antecedents and psychobehavioral consequences, it is important to quantify both “state anxiety” and “trait anxiety.” “State anxiety” is defined as a temporary reaction to adverse events, and this emotional state is supposed to be more transient and intense than “trait anxiety.” “State anxiety” is associated with temporarily increased sympathetic nervous system activity, while “trait anxiety” is a relatively constant feature of personality (29, 30). Some evidence suggests that state and trait anxiety might be multidimensional constructs not closely associated with each other (31). Therefore, the effects of yoga on anxiety should be studied by focusing on both state and trait anxiety, as these anxiety parts represent different sides of the same “coin.” The Spielberger State-Trait Anxiety Inventory (STAI) is one of the most frequently exploited approaches to studying anxiety because of its reliable and sensitive measure of anxiety and the significant superiority of STAI over other approaches to measuring anxiety (32).

Yoga is supposed to serve as a platform for various physical, mental, and spiritual practices originating thousands of years ago in India (33, 34). Yin yoga is less known in the Western culture than hatha yoga. Yin yoga is more meditative, slower, and more focused on calmness and mindfulness than hatha yoga (35). Yin and yang are fundamental terms associated with the Chinese philosophy of complementary forces. A yin yoga sequence consists of a series of passive yoga poses (asanas) typically held between three to ten minutes (36, 37). Yin yoga asanas are supposed to stretch tissues called the yin tissues. Yin tissues are the tendons, fascia, ligaments, and the other connective tissues of the body. In yin yoga sequences, the use of muscles is kept to a minimum compared with more dynamic yang yoga (e.g., hatha yoga). The emphasis is on relaxed belly breathing. Taking long, slow, and deep breaths allows the human body to relax, enabling it to stay in an asana for a longer duration of time. Thus, yin yoga focuses on a relatively passive approach and works more on the physical level with the body’s connective tissues, ligaments, tendons, bones, and joints (fascial stretching), including the hip, pelvis, knee, and spinal joints, rather than the muscles (37). The number of asanas or postures in yin yoga is much lower than in hatha yoga (36). Yin yoga focuses on breathing and diaphragmatic breathing (belly breathing), which activates the parasympathetic nervous system and signals the body to relax and unwind. The main foci of yin yoga are awareness and concentration on the breath, or staying static in a posture without excessive movement, allowing gravity to act on the body (38). Because of combining sequences of physical postures with deep breathing in a controlled, slow, and mindful manner, yin yoga has been suggested to have tremendous potential to lower perceived stress and anxiety levels (33, 36).

As yoga offers a popular and promising treatment method for anxiety with a poorly understood mechanism (33, 36, 39–41), this study investigates whether yin yoga significantly reduces situational anxiety immediately after each treatment and at the end of the 10-week yoga intervention compared with the beginning of the study. In contrast, we predicted no effect of yin yoga intervention on trait anxiety because theoretically, trait anxiety is a stabler part of personality. It should, however, be noted that a prior study found that participation in a single yin yoga session reduced both state anxiety and trait anxiety (39).

## 2 Material and methods

### 2.1 Research design and participants

**Figure 1:**
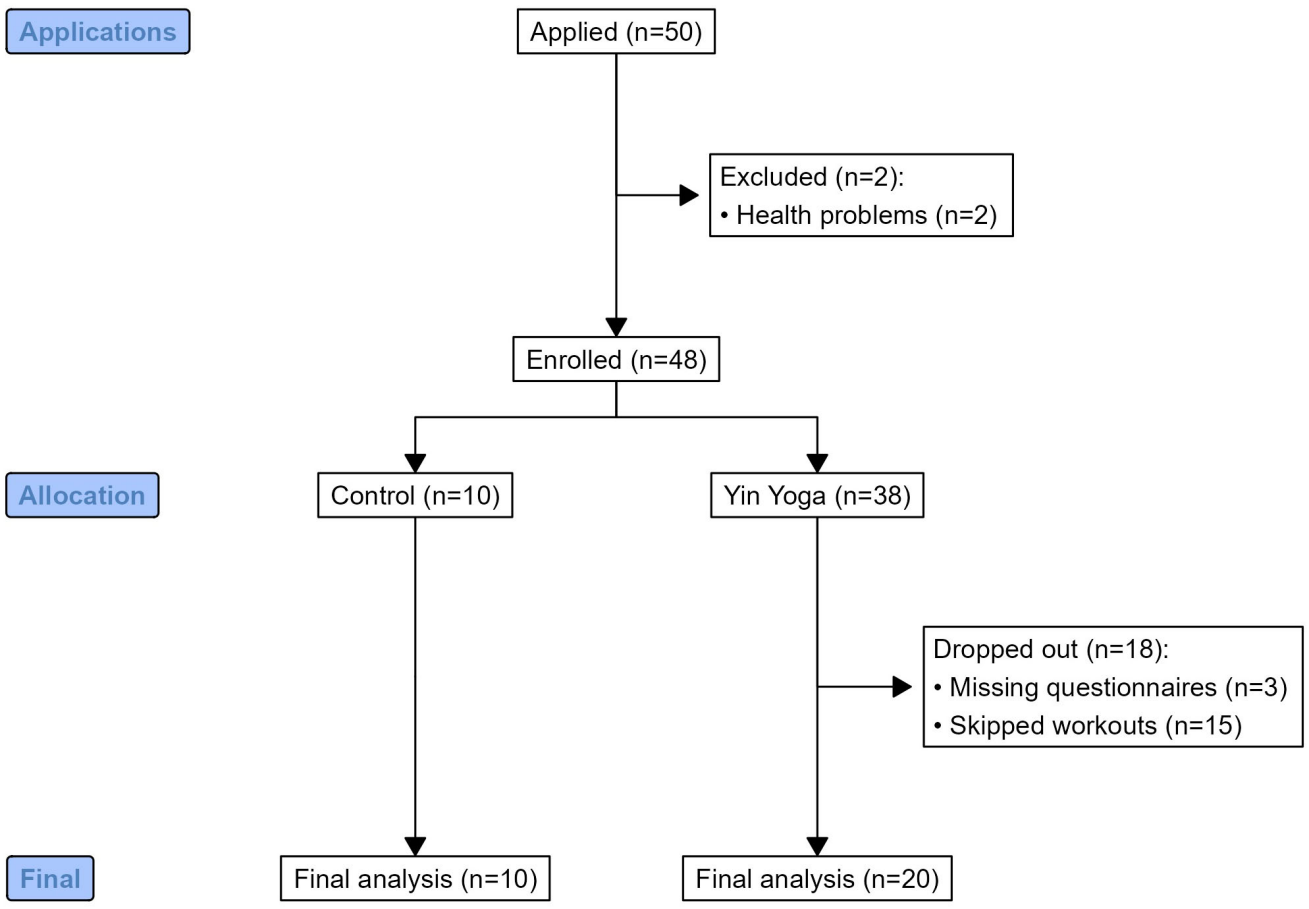
The flowchart illustrating the research design and participant enrolment.

The study was conducted in Latvia between September and November 2021 during the COVID-19 pandemic. We followed the STROBE (Strengthening the Reporting of Observational Studies in Epidemiology) statement and recommendations while planning and designing this study (42) (**Figure 1**). Our yoga expert (KS) had ten years of experience in yoga teaching at the time of conducting this study. This study was performed as a part of her study project in the Health and Sports Program of Riga Stradins University (the leading medical university of Latvia). This study was carried out via Zoom, which prevented the SARS-CoV-2 virus from spreading among participants. The participants of the study were women aged 22-55 (mean = 34.44 ± 5.86). All the participants experienced psycho-emotional disorders during the pandemic, including anxiety, confusion, or other depression-like emotions. None of the participants was diagnosed with clinical depression, posttraumatic stress disorder, eating disorders, substance use disorders, significant suicidal ideation, bipolar disorder, schizophrenia, or developmental disorders. The participants had never been infected with the SARS-CoV-2 virus before or during the study. Participants were not taking any prescription medication for at least 1 month. None of the participants had completed more than 5 yoga or cognitive therapy sessions (CBT) sessions in the past 5 years.

The participants were recruited through an advertisement posted on social media (Instagram), which clearly explained the purpose of the study. It was explained that there will be a study that requires active working women who have no experience in yin yoga (or in any other form of yoga) and who have experienced anxiety symptoms repeatedly and regularly, at least once a week, in the last 6 months. Some participants had been to the doctor and been diagnosed with an anxiety disorder, but had not started drug treatment. The study participants had such anxiety symptoms as insomnia, overthinking, nervousness, migraines, digestive problems, dizziness, vomiting, shortness of breath, heart problems, and panic attacks. All participants received detailed information and instructions on how to participate in the study. Within two consecutive days, 50 applications for participation were received, from which 38 participants were randomly selected to participate in the yoga intervention group, and 10 participants were included in the control group. The study did not include two participants because of health problems (**Figure 1**). All participants were new to yoga and were invited to participate in the control group or the intervention group. Since the inclusion criteria of the study were quite numerous and strictly defined, it was important that the intervention group had a sufficiently large number of participants. During the COVID-19 pandemic, there was a high possibility of dropout of study participants. Therefore, from the very beginning, a larger number of participants in the intervention group than in the control group was planned, so that in the event of participants dropping out, the size of the intervention group would not decrease too critically. The participants were randomly assigned to groups and were never transferred from one group to another. None of the participants expected to be in the intervention group but ended up in the control group and vice versa.

### 2.2 Working environment and anxiety estimation before the study

The Spielberger Anxiety Self-Assessment Questionnaire (STAI) was used to evaluate participants’ anxiety levels both before the study and during the study; only participants with anxiety symptoms were included in this study (32). An important requirement for the subjects to be included as participants in this study was their occupational status: all participants were employed by businesses of different sizes, and all participants worked remotely during the study. Two participants combined their work with university studies. The income level of the participants was estimated as average according to Latvian standards. Thus, income and working status were considered similar across the sample.

Although the presence of anxiety symptoms was not among the inclusion criteria for the study, all participants reported some anxiety-related feelings, such as feeling afraid or easily irritable (13, 43), at the beginning of the study. The participants reported that their anxiety arose primarily from social distancing policies and especially from their remote work.

### 2.3 Brief characterization of yin yoga

A typical yin yoga class differs from a classical yoga class in its pace. Yin yoga is slower, and each pose is usually performed for 3-10 minutes. The emphasis is not on muscle stretching or tension but, on the opposite, which is muscle relaxation. Yin yoga aims to activate the parasympathetic nervous system to reach the deeper layers of the fascia and keep the joint open. In classical yoga, the muscles are tensed to close the joint. In yin yoga, there are no inverted poses, no balancing poses, and no standing positions. In yin yoga, everything is done sitting or lying down, with only a few exceptions. Classical yoga has three times as many poses because they must be held for a relatively shorter time, and the classes are generally more dynamic than in yin yoga. A typical yin yoga class begins with a short meditation, focusing on the breath, perhaps with one of the breathing exercises. For the effect of yin yoga to reach the layers of the deep fascia, the muscles should preferably be cold, so there is no need to warm up before a yin yoga class.

### 2.4 Yoga poses (asanas)

The sequence of yin yoga asanas in this study was the following in each yoga lesson: Butterfly with forward bend (6 min), Deer (10 min, 5 min on each side), Square (8 min, 4 min on each side), Squat (6 min), Child (8 min), Shoelace (10 min, 5 min on each side), Dragon (12 min, 6 min on each side), Swan (10 min, 5 min on each side), Downward facing dog (1-2 min), Half Saddle (8 min), Snail (5 min), and Savasana (Corpse pose) (15 min) (37). There was usually a 30-second break between yoga asanas, allowing participants to move from one pose to the next. The final relaxation at the end of each yoga lesson lasted for 10-12 min. The duration of each pose slightly varied individually as some participants sometimes could not hold some of the poses for the maximum duration, especially at the beginning of the study.

### 2.5 Study protocol

While participants in the intervention group were supposed to perform yin yoga exercises for the duration of 10 weeks, participants in the control group agreed not to change anything in their lifestyle for the 10 weeks of the study. The participants in the experimental group had yoga classes once a week. The classes were held on the Zoom platform with the option of recording the class. The yoga teacher (KS) continuously monitored the quality of yoga exercises performed by study participants on the computer screen. Each class was available as a recording for one week, after which it was deleted. The duration of the classes was 120 min. Each online class was held on Thursday evenings from 18:30 to 20:30 or on Friday mornings from 9:00 to 11:00. Each class consisted of breathing exercises, meditation, and fascial stretching exercises.

### 2.6 Assessment of state and trait anxiety

The Spielberger Anxiety Self-Assessment Questionnaire (STAI) consists of two parts (STAI Y-1 and STAI Y-2) and provides self-assessment indicators of state anxiety (STAI Y-1) and trait anxiety (STAI Y-2) (32). The participants of the intervention group completed both parts of the questionnaire before and after each yoga class, and these participants filled both questionnaires also before the study and after the study exactly as done by the participants of the control group. In contrast, the control group did not participate in the yoga classes. The participants of the control group completed both parts of the questionnaire STAI Y-1 and STAI Y-2 only twice: before the start and at the end of the study.

“State-Trait Anxiety Inventory, Form Y; Self Evaluation Questionnaire” has been developed by Spielberger et al. (32), and adapted into Latvian by (44, 45). The STAI Questionnaire has been adapted into more than 46 languages for cross-cultural research and clinical practice. The questionnaire consists of two parts; the first part, STAI Y-1, comprises 20 questions and determines the level of anxiety at a specific moment (state anxiety). The second part of the questionnaire, STAI Y-2, also consists of 20 questions that measure how a person usually feels, determining the individual’s anxiety state or anxiety as a feature level (trait anxiety), that is, how anxious a person is in everyday life. Questionnaires consist of positive and negative statements, each with four possible answers. In each of the questions, the participant can get between one and four points, a total of 20-80 points for the first part of the questionnaire. The same number of points can be gathered for the second part of STAI questionnaire. Each part of the questionnaire is evaluated separately. The higher the number of points, the greater the anxiety at the time, and anxiety as a trait of a person’s character. The first part of the questionnaire provides positive and negative statements that people use to describe their feelings right now, at this moment. For example: “I feel anxious; I feel excited; I feel calm; I feel easy.” Possible answers are: “No, not at all; a little bit; rather yes; yes, certainly.” The second part of the questionnaire contains statements people use to describe their typical feelings they experience daily. For example: “I feel nervous; I feel like a failure; I get stressed when I think about my worries; I feel safe; I make decisions easily; I am a balanced person.” Possible answers include: “No, not at all; a little bit; rather yes; yes, certainly.”

STAI Y-1 (state anxiety) is a feeling of worry, tension, nervousness, and anxiety. In addition to assessing how people feel “right now,” the STAI Y-1 can also be used to determine how they felt at a specific time in the recent past and how they anticipate they will feel in a particular situation that is possible and that they may face in the future or in various hypothetical situations. State anxiety scores increase in response to physical danger and psychological stress and decrease in relaxation training. The STAI Y-1-Anxiety Scale is a sensitive measure of changes in transient anxiety experienced by patients/clients in counseling, psychotherapy, and behavior modification programs. The scale is widely used to assess the level of anxiety caused by stressful experimental procedures and imminent real-life stressors, such as imminent surgery, a dental visit, a job interview, or important school tests (32).

The STAI Y-2 anxiety scale (trait anxiety) is widely used to assess clinical anxiety in medical, surgical, psychosomatic, and psychiatric patients (46). Psychoneurotic and depressed patients usually have high scores on this scale. This anxiety scale is also used to screen for anxiety among high school and college students and military personnel and to evaluate immediate and long-term outcomes of psychotherapy, counseling, behavior modification, and substance abuse treatment programs (47). In clinical and experimental studies, the STAI Y-2 scale helps identify individuals with high levels of neurotic anxiety and for selecting participants with different motivations or levels of motivators for psychological experiments (32). Importantly, STAI Y-2 (trait anxiety questionnaire) is more often used in research than the STAI Y-1 because the state anxiety detected by STAI Y-1 depends on various external factors. STAI Y-2 describes the psycho-emotional state of a person in a more general way than STAI Y-1.

There were no time limits for filling the STAI Y-1 and Y-2 questionnaire parts. On average, one part of the questionnaire takes 6 minutes, and both parts usually take c. 10 minutes to fill in. For emotional participants, completing the questionnaire may take up to 20 minutes.

### 2.7 Statistical analyses

To explore differences between anxiety states before and after the study in control and experimental groups, we fitted two linear mixed-effects models using lme, setting STAI Y-1 and STAI Y-2 as dependent variables. The treatment group (control or experimental) and time of the measurement (before and after the study) were set as fixed effects. In addition, the unique identifier of each participant was set as random effects to account for repeated measurements. We also performed *post-hoc* pairwise comparisons using Tukey’s honestly significant difference (HSD) method to account for multiple comparisons. We fitted a linear mixed effects model to assess how STAI Y-1 differed between the beginning and end of each workout in the study and how STAI Y-1 changed as the study progressed. We set STAI Y-1 as the dependent variable, and workout number (1 to 10) and measurement time (before or after each workout) as fixed effects. We also fit two additional models: setting STAI Y-1 before the workout as the dependent variable in one of the models and STAI Y-1 after each workout as the dependent variable in the second model. We set the number of workouts as the independent variable and the unique identifier of each participant as a random variable to account for repeated measures and individual variability. Residuals in all models were tested for the assumption of normality and homoskedasticity graphically using histograms of residuals and scatterplots of residuals against predicted values. All statistical analyses were conducted in R software, version 4.1.0. Linear mixed-effects models were fitted using “lme4” package, assessing the statistical significance and calculating *P* values using the function “Anova” from “car” package. *Post-hoc* tests were performed with “emmeans” package. We conducted all statistical tests at the significance level of *P* < 0.05.

## 3 Results

### 3.1 State anxiety (STAI Y-1) before and after the study

Linear mixed-effects model showed significant differences in STAI Y-1 scores as a function of group identity (control vs experimental) (χ2 = 9.51, df = 1, *P* < 0.001), time (the beginning vs the end of the study) (χ2 = 8.04, df = 1, *P* < 0.01), and their interaction (χ2 = 6.85, df = 1, *P* < 0.01).

**Figure 2:**
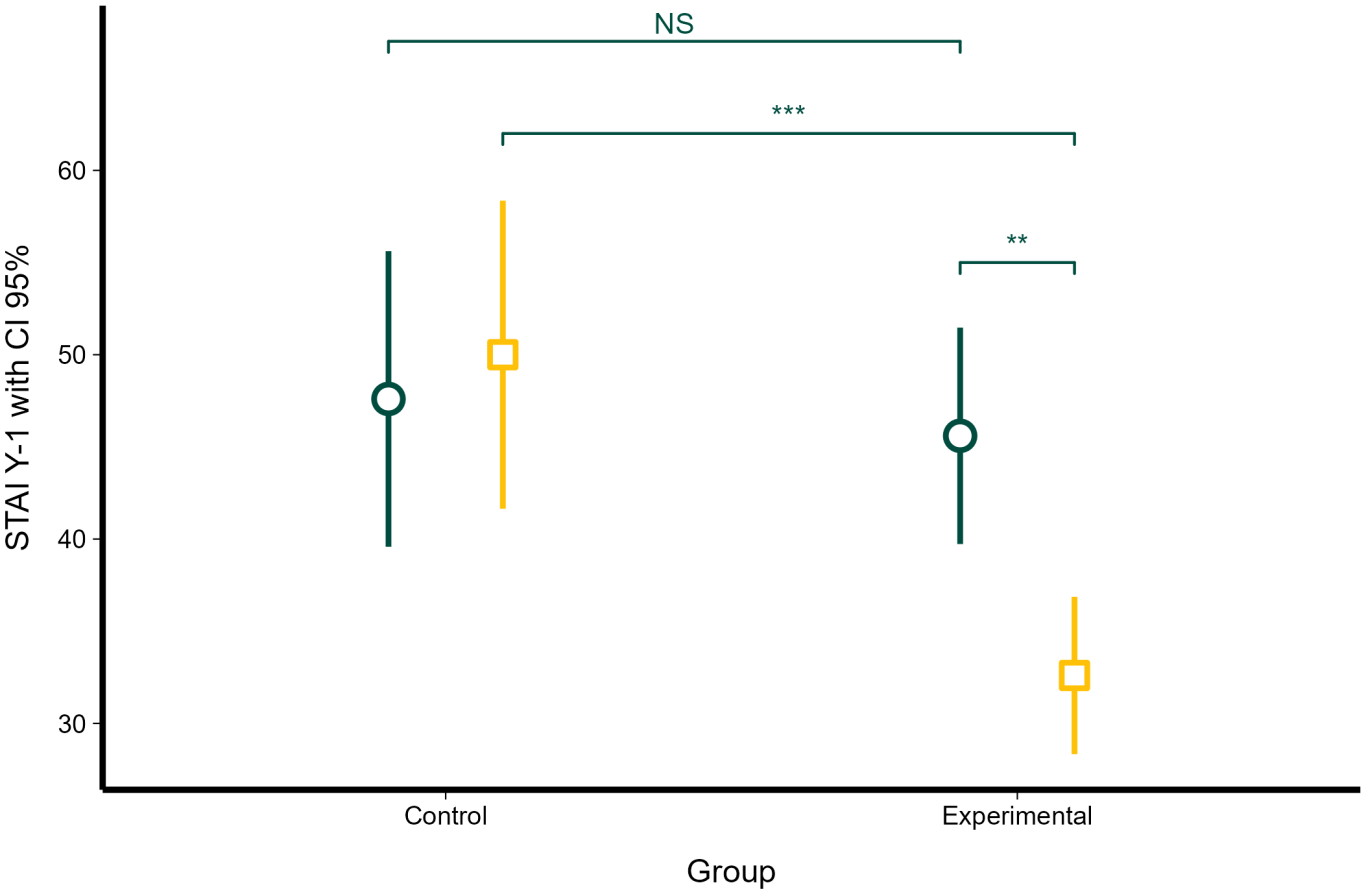
Changes in state anxiety (STAI Y-1) in the control and intervention groups before the study and at the end of the study. Dark green circles represent STAI Y-1 scores at the beginning of the study; dark yellow squares represent STAI Y-1 at the end of the study. Error bars represent 95% confidence intervals. Statistical significance, as obtained with linear mixed-effects model, is denoted as non-significant (NS), *P* < 0.01 (**), and *P* < 0.001 (***).

STAI Y-1 of the control group (mean = 47.60, *SE* = 3.52) and experimental group (mean = 45.60, *SE* = 2.49) did not differ significantly at the beginning of the study (*P* = 0.967, **Figure 2**). At the end of the study, STAI Y-1 did not significantly change during the ten-week period in the control group (mean = 47.60 and *SE* = 3.52 vs. mean = 50.00 and *SE* = 3.52; *P* = 0.97; **Figure 2**), while STAI Y-1 significantly decreased in the intervention group (mean = 45.60 and *SE* = 2.49 vs. mean = 32.60 and *SE* = 2.49; *P* = 0.0035; **Figure 2**). STAI Y-1 of the intervention group was significantly lower than in the control group at the end of the study (*P* = 0.0009; **Figure 2**).

### 3.2 Trait anxiety (STAI Y-2) before and after the study

Linear mixed-effects model showed that time (the beginning vs. the end of the study) (χ2 = 83.22, df = 1, *P* < 0.001) and interaction between time and group (χ2 = 99.38, df = 1, *P* < 0.001) significantly affected STAI Y-2. However, group identity (control vs experimental) did not have a significant effect on STAI Y-2 (χ2 = 0.012, df = 1, *P* = 0.912).

**Figure 3:**
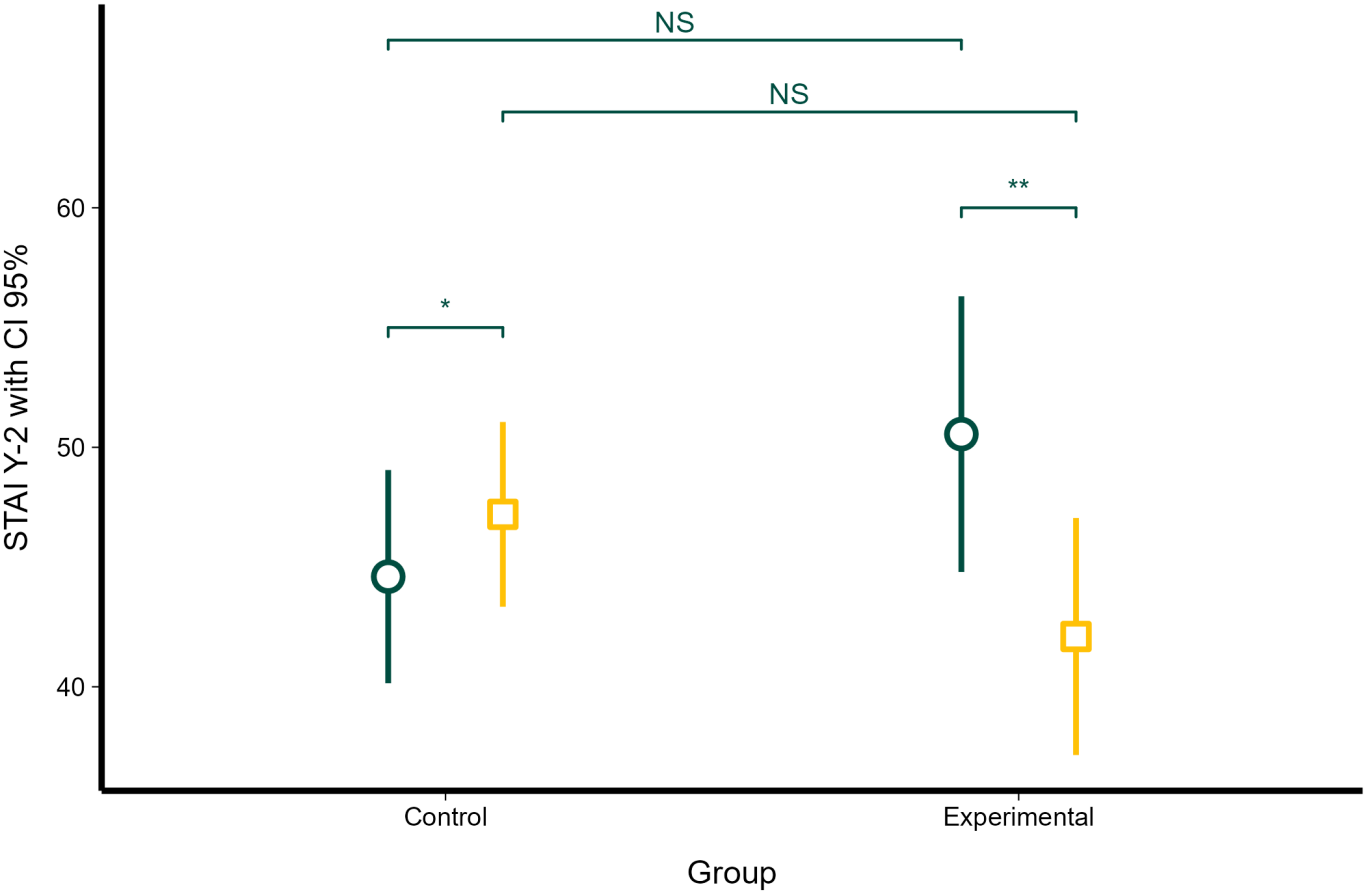
Changes in trait anxiety (STAI Y-2) in the control and experimental groups at the beginning of the study and at the end of the study. Dark green circles represent STAI Y-2 scores at the beginning of the study; dark yellow squares represent STAI Y-2 at the end of the study. Error bars represent 95% confidence intervals. Statistical significance, as obtained with linear mixed-effects model, is denoted as non-significant (NS), *P*, < 0.05 (*) and *P* < 0.01 (**).

The control and experimental groups did not significantly differ at the beginning of the study in STAI Y-2 scores (mean = 44.60 and *SE* = 3.16 vs. mean = 50.50 and *SE* = 2.24; *P* = 0.43; **Figure 3**). STAI Y-2 significantly increased between the beginning and the end of the study in the control group (mean = 44.60 and *SE* = 3.16 vs. mean = 47.20 and *SE* = 3.16; *P* = 0.036; **Figure 3**), and STAI Y-2 significantly decreased during the study in the intervention group (mean = 50.50 and *SE* = 2.24 and mean = 42.10 and *SE* = 2.24; *P* < 0.0001; **Figure 3**). Despite these opposite changes in STAI Y-2, the control group and the intervention group did not differ at the end of the study (mean = 47.20 and *SE* = 3.16 vs. mean = 42.10 and *SE* = 2.24; *P* = 0.56; **Figure 3**).

### 3.3 STAI Y-1 before and after each yoga workout

**Figure 4:**
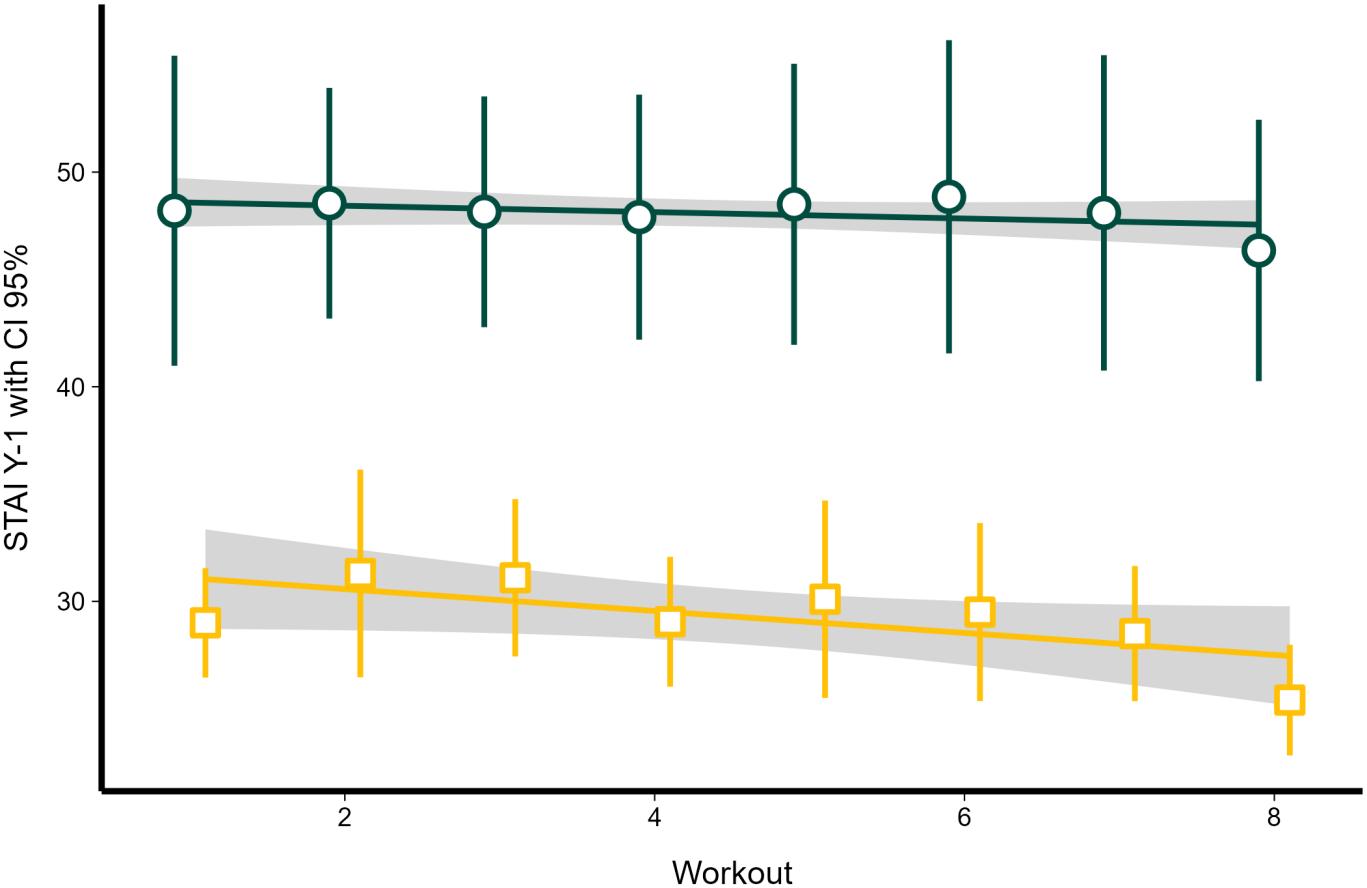
The dynamics of STAI Y-1 before and after each yin yoga class in the treatment group. Regression lines obtained with linear mixed-effects models. Dark green circles represent STAI Y-1 scores before each workout; dark yellow squares represent STAI Y-1 after each workout. Error bars and grey area represent 95% confidence intervals.

Linear mixed-effects model showed that STAI Y-1 significantly differed between the beginning and the end of each yoga workout (χ2 = 531, df = 1, *P* < 0.0001; **Figure 4**), while it did not significantly differ between different workouts (χ2 = 8.35, *P* = 0.30). STAI Y-1 was not affected by the workout number during the study, nor by the interaction between workout number and time of assessment (before and after a yoga workout) (χ2 = 2.13, df = 7, *P* = 0.95).

STAI Y-1 before each workout also showed no significant relationship with workout number (χ2 = 0.87, df = 1, *P* = 0.351; **Figure 4**). However, post-workout STAI Y-1 showed a significant decrease as the workouts progressed (χ2 = 6.71, df = 1, *P* < 0.001; **Figure 4**), decreasing by 0.51 (*SE* = 0.197) units with each yin yoga class.

We did not observe any adverse effects of yin yoga in this study.

## 4 Discussion

A large number of studies have shown an increase in mental health issues owing to the COVID-19 pandemic (13, 48–50). General distress and reduced social contacts, including remote work, had detrimental effects on mental health, increasing stress, anxiety, and depression. The results of this study showed that yoga practice during the COVID-19 pandemic resulted in lower levels of anxiety. The findings are relevant in such unusual occupational contexts where employees are forced to work remotely owing to public health concerns arising from an epidemic, a pandemic, or other hazards.

The results of this study show that (i) a 10-week yin yoga treatment delivered online significantly reduced state anxiety (STAI Y-1) in the intervention group compared with the control group; (ii) yoga exercises did not cause differences in trait anxiety (STAI Y-2) between the control and intervention groups, even if it decreased trait anxiety in the intervention group, and (iii) state anxiety levels decreased significantly after each yin yoga class in the intervention group, but did not change in the control group. Overall, this study demonstrates the positive effects of yin yoga on state anxiety. Importantly, this study shows both short-term and long-term effects of yin yoga on anxiety, suggesting yin yoga intervention as a first-line treatment to control and reduce state anxiety. With somewhat larger samples, these results support those of an earlier study (39).

In contrast to the positive effects found in this study, other studies on yoga intervention on anxiety have showed mixed results. For example, a meta-analysis (51) showed that hatha yoga was not practical for treating anxiety disorders. Simon et al. (23) found that Kundalini yoga was not as effective as the CBT for the treatment of generalized anxiety disorder (52), which does not suggest Kundalini yoga as a first-line treatment to reduce anxiety. On the other hand, a meta-analysis (22) showed that yoga exercises can effectively decrease anxiety levels but only for participants with elevated anxiety. Cramer et al. (22) concluded that yoga might be an effective intervention for individuals with anxiety. As Cramer et al. (22) and Vollbehr et al. (51) suggested, the results of their meta-analyses are mixed and often inconclusive because of the lack of rigorous research designs. However, another systematic review (53) found that yoga was effective regardless of the sample size or research design. Importantly, unlike other meta-analyses, this review article excluded such search terms as depression because anxiety and depression are frequently comorbid and studied together. The credibility of the review by Laban-Sharman et al. (53) was increased by including studies with validated anxiety assessment scales, such as State–Trait Anxiety Inventory and similar rating scales. The only exception was a study by Simon et al. (23) using a Clinical Global Impression of Improvement scale. The State-Trait Anxiety Inventory approach probably made finding positive effects of the 10-week yin yoga practice possible. Therefore, we suggest using measures such as State–Trait Anxiety Inventory or similar (Beck Anxiety Inventory, Hamilton Anxiety Rating Scale) for future research on yoga efficacy.

Interestingly, this study found a significant effect of yin yoga in a relatively small sample of participants, which replicates earlier findings (40, 54) from studies that also had small sample sizes. The first reason for our ability to confirm the positive effects of yin yoga on the reduction of anxiety symptoms is the already mentioned State-Trait Anxiety Inventory approach, which allowed us to dissect participants’ mental states in more detail. As this study was performed during the COVID-19 pandemic, an unprecedently high level of anxiety might be another reason for the observed positive effect of the treatment of yin yoga. The COVID-19 pandemic had a long-term impact on people worldwide (55). Restrictive epidemiologic measures, mortality, morbidity, social isolation, and separation affected the mental state of people worldwide (11, 56). Numerous risk factors lowered predictability in life, which increased stress levels, leading to several behavioral psychopathologies mediated by changes in brain structure that are associated with severe anxiety and depression (57, 58). Therefore, activities like the yoga intervention used in the current study might have positively added to the frequency of social interactions as well as increasing the sense of individual worth, personal identity, and meaningfulness of life, potentially repairing security-enhancing social relationships that had eroded during the pandemic (59). Nevertheless, because this study included only a yoga intervention (and a control group that received no intervention), we cannot confirm that the anxiety-reducing effects of such an exercise intervention pertain solely to yoga, or whether they would be present in any physical activity done either in a group or alone. For instance, studies have shown that other exercise modalities besides yoga have been effective at reducing anxiety during the COVID-19 pandemic, including exergames (which are based on the combination of exercise with appealing digital games) as well as other types of physical exercise (60). Other limitations of this study include the fact that it included only women living in Latvia, limiting the generalizability of the findings. It has been shown that the COVID-19 pandemic significantly increased anxiety levels in the Latvian population (e.g., 61). However, we did not observe a significant difference in the trait anxiety of the participants between the control and the treatment groups at the beginning and the end of the study despite some fluctuations of an opposite character in each group. In contrast to state anxiety, trait anxiety constitutes a suite of personality traits describing individual differences. Therefore, trait anxiety is considered to be relatively stable over short periods (27, 62, 63), and it was surprising that trait anxiety increased in the control group and decreased in the intervention group over the study period.

Based on personal experience gained during this study, we suggest that the quality of yoga teachers/instructors might cause mixed or inconsistent results found on the associations between yoga and anxiety in many previous studies. Although the quality of yoga teachers has not been considered a crucial factor in reaching credible results in most studies, (64) showed a general lack of awareness of yoga research among most yoga teachers and therapists, potentially affecting the quality of their yoga practice. While most yoga instructors agree that yoga research is important, only a few seriously update themselves on this through scientific literature. This demonstrates that yoga instructor quality must be considered as a random factor that can contribute to possible discrepancies between the results of yoga studies (65).

Recent research has found a possible mechanism to explain yoga’s benefits to the body (66). Exercise induces a four-to five-fold increase in the amount of blood pumped out by the heart, which requires faster heartbeats and more forceful contractions of heart muscles (67). The heart’s ability to pump blood is modulated by nerves called ‘autonomic’ since they work automatically and do not require conscious effort. These nerves include the ‘fight or flight’ or ‘sympathetic’ nerves and the ‘rest and digest’ vagal nerves, termed ‘parasympathetic’ (68). The vagal nerve connects the brain to the heart and other internal organs, including the gut, regulating the ‘rest and digest’ parasympathetic nervous system responses. Recent research has found that the parasympathetic and sympathetic nervous systems work together in exercise to help the heart pump harder and faster. Shanks et al. (66) showed that parasympathetic nerves dilate the coronary vessels by neuromodulator vasoactive intestinal peptide, allowing more blood to pump through the heart. Since yoga can improve the condition of the parasympathetic nervous system (69), it can reduce anxiety and the health condition of the entire body. This requires more research on yoga while considering both parasympathetic and sympathetic nervous systems to test whether relaxation and regular exercise can improve vagal activity and how the beneficial effects are reached (69).

This study was limited by social distancing regulations during the COVID-19 pandemic. Therefore, the social connection and mutual influence of the study participants and the yoga teacher were indirect. The in-person contact between study participants and the yoga teacher may have been much closer if the study was conducted outside a pandemic scenario in an in-person yoga studio. Despite the lack of direct personal contact, yoga classes were able to reduce anxiety among study participants, indicating a positive effect of yoga on participants’ perceived stress levels. Finally, modern lifestyle, in general, and the COVID-19 pandemic, in particular, restricted the natural range of locomotory and aerobic activities of most people. Walking, running, and other forms of aerobic activity help reduce excess body fat, which causes low-grade inflammation in the CNS, worsens physical and mental health, and even prevents the formation of new brain cells and connectivity between brain cells (70, 71). As our results show significant anti-anxiety effects of yin yoga, a potential further avenue of research is to study possible anti-aging effects of yoga on the human nervous system.

## 5 Conclusion

To conclude, the results of this study show that yoga participation significantly reduced state anxiety (STAI Y-1) and trait anxiety (STAI Y-2) in women. However, conditions of the social environment (such as the stress, anxiety, and unpredictability caused globally by the COVID-19 pandemic) and probably also the quality of yoga instructors should be considered while analyzing the efficacy of yoga on anxiety. Overall, the results suggest that yoga, even when delivered online, can be considered as a cost-effective intervention that can address some psychological aspects of occupational hazards, promoting mental health in a remote work occupational setting as well as improving overall quality of life and well-being through reduced anxiety.

## Data availability statement

The raw data supporting the conclusions of this article will be made available by the authors, without undue reservation.

## Ethics statement

The studies involving humans were approved by The Ethical Committee of the Riga Stradins University. The studies were conducted in accordance with the local legislation and institutional requirements. The participants provided their written informed consent to participate in this study. Written informed consent was obtained from the individual(s) for the publication of any potentially identifiable images or data included in this article.

## Author contributions

KS: Conceptualization, Data curation, Formal analysis, Investigation, Methodology, Visualization, Writing – original draft, Writing – review & editing. MM: Formal analysis, Software, Visualization, Writing – original draft, Writing – review & editing. RK: Resources, Software, Supervision, Validation, Visualization, Writing – review & editing. GR: Conceptualization, Formal analysis, Funding acquisition, Validation, Writing – review & editing. SL: Conceptualization, Formal analysis, Validation, Writing – review & editing. IK: Conceptualization, Data curation, Project administration, Resources, Supervision, Writing – original draft.
