# Supplementary material for: The effect of yin yoga intervention on state and trait anxiety during the COVID-19 pandemic
Source: Front Psychiatry. 2024 Mar 14;15:1345455. doi: 10.3389/fpsyt.2024.1345455 (PMC10973109; doi:10.3389/fpsyt.2024.1345455)
Supplement: Supplementary file 1 [file DataSheet_1.docx]

**The qualifications of the yoga teacher (KS) include the following national and international certificates:**

**1) International Yoga Healing Federation, certificate 001/A110: “Yoga Therapy”, 2008**

**2) International Yoga Healing Federation, certificate 001/ A210: “Yoga”**

**3) Higher Interregional Attestation Committee, certificate 00028: “Yoga specialist (professional)”, 2013**

**4) Yoga Education institution, Certificate in Children’s yoga teacher training, 2014**

**5) “With yin yoga”: Yin yoga teacher Certificate (100 h), 2015**

**6) Riga Stradins University certificate of professional development: "Fundamentals of fitness – pre-pilates and pilates methods – Level 1", 13 h, 2016**

**7) Latvian Coaches Continuing Education Center: “A healthy back for a quality life”, 8 h, 2016**

**8) Riga Stradins University certificate of professional development: "Pilates method exercises for an average fitness level (level 2), 13 h, 2016**

**9)  Riga Stradins University certificate of professional development: Possibilities of using small equipment in pilates classes, 7 h, 2017**

**10) Latvian Coaches Continuing Education Center: “Dynamic anatomy during training process”, 8 h, 2017**

**11) Latvian Academy of Sports Education: “Mindfulness and mental resilience as a means of increasing the effectiveness of the training process and athletic results in high performance sports”, 8 h, 2018**

**12) Latvian Federation of Bodybuilding, Fitness and Body fitness: "improvement of qualification of a fitness trainer”, 8 h, 2018**

**13) Latvian Sports Education Agency: C-category fitness trainer, 2018 (9-month education course)**

**13) Swayambhu Adi yoga foundation (India): 2019, 7 days (100 h) yoga teacher qualification course.**

**14) Latvian Coaches Continuing Education Center: “Pilates with small equipment”, 20 h, 2021**

**15) Riga Stradins University: Health Sport Specialist; Fitness instructor qualification, B level, 2022**
